# PEDF promotes the repair of bone marrow endothelial cell injury and accelerates hematopoietic reconstruction after bone marrow transplantation

**DOI:** 10.1186/s12929-020-00685-4

**Published:** 2020-09-01

**Authors:** Wen Ju, Wenyi Lu, Lan Ding, Yurong Bao, Fei Hong, Yuting Chen, Hui Gao, Xiaoqi Xu, Guozhang Wang, Weiwei Wang, Xi Zhang, Chunling Fu, Kunming Qi, Zhenyu Li, Kailin Xu, Jianlin Qiao, Lingyu Zeng

**Affiliations:** 1 Blood Diseases Institute, Xuzhou Medical University, Xuzhou, China; 2 Key Laboratory of Bone Marrow Stem Cell, Jiangsu Province, Xuzhou, China; 3 Department of Hematology, the Affiliated Hospital of Xuzhou Medical University, Xuzhou, China; 4 Medical Center of Hematology, Xinqiao Hospital, Third Military Medical University, Chongqing, China

**Keywords:** PEDF, Endothelial cells, Hematopoietic stem cells, Hematopoietic reconstitution

## Abstract

**Background:**

Preconditioning before bone marrow transplantation such as irradiation causes vascular endothelial cells damage and promoting the repair of damaged endothelial cells is beneficial for hematopoietic reconstitution. Pigment epithelium-derived factor (PEDF) regulates vascular permeability. However, PEDF’s role in the repair of damaged endothelial cells during preconditioning remains unclear. The purpose of our study is to investigate PEDF’s effect on preconditioning-induced damage of endothelial cells and hematopoietic reconstitution.

**Methods:**

Damaged endothelial cells induced by irradiation was co-cultured with hematopoietic stem cells (HSC) in the absence or presence of PEDF followed by analysis of HSC number, cell cycle, colony formation and differentiation. In addition, PEDF was injected into mice model of bone marrow transplantation followed by analysis of bone marrow injury, HSC number and peripheral hematopoietic reconstitution as well as the secretion of cytokines (SCF, TGF-β, IL-6 and TNF-α). Comparisons between two groups were performed by student t-test and multiple groups by one-way or two-way ANOVA.

**Results:**

Damaged endothelial cells reduced HSC expansion and colony formation, induced HSC cell cycle arrest and apoptosis and promoted HSC differentiation as well as decreased PEDF expression. Addition of PEDF increased CD144 expression in damaged endothelial cells and inhibited the increase of endothelial permeability, which were abolished after addition of PEDF receptor inhibitor Atglistatin. Additionally, PEDF ameliorated the inhibitory effect of damaged endothelial cells on HSC expansion in vitro. Finally, PEDF accelerated hematopoietic reconstitution after bone marrow transplantation in mice and promoted the secretion of SCF, TGF-β and IL-6.

**Conclusions:**

PEDF inhibits the increased endothelial permeability induced by irradiation and reverse the inhibitory effect of injured endothelial cells on hematopoietic stem cells and promote hematopoietic reconstruction.

## Background

Hematopoietic stem cell transplantation (HSCT) is widely used for treating hematological malignancies [1–3]. However, long-term hypoglycemia after transplantation, that is, poor graft function (PGF), seriously affects patient survival and quality of life [4, 5]. Studies have shown that PGF is closely related to the hematopoietic microenvironment [6]. Therefore, in-depth exploration of microenvironmental factors affecting HSC homing and implantation, and accelerating hematopoietic reconstruction and hematopoietic function recovery after HSCT are potential research directions in the field of HSCT transplantation.

Hematopoietic microenvironment is an internal environment that regulates and supports the growth and development of hematopoietic cells. It is mainly composed of stromal cells and extracellular matrix [7, 8]. Among them, endothelial cells are an important part of the hematopoietic microenvironment and involved in hematopoietic reconstruction [9–11]. Our previous study found that infusion of endothelial progenitor cell (EPC) can reduce the incidence and severity of graft-versus-host disease (GVHD) and promote hematopoietic reconstruction after HSCT [12]. EPC can differentiate into endothelial cells and promote the repair of injured vascular niche, indicating its important role in hematopoietic reconstruction [13]. Under normal conditions, mature vascular endothelium is in a stable quiescent state, but under pathological conditions, the vascular endothelium is detached, leading to morphology changes, increased vascular permeability and vascular fibrosis [14–16]. We previously found that preconditioning regimens prior to HSCT could cause damage to vascular endothelial structure and function and changes in endothelial permeability [17–19]. However, the current underlying mechanisms of endothelial injury and strategies to promote endothelial repair during preconditioning treatment are still lacking [14].

Pigment epithelium-derived factor (PEDF) is a 50-kDa non-inhibitory factor in the serine protease inhibitor gene family and secreted by vascular endothelial cells, pericytes and retinal pigment epithelial cells [20]. Several studies have shown that PEDF is closely related to the function of vascular endothelial cells and exerts multiple effects such as anti-inflammation, antioxidant, anti-tumor, anti-angiogenesis, and inhibition of vascular permeability [21–25]. PEDF can inhibit vascular endothelial growth factor (VEGF) and stress-induced increase in vascular permeability in vitro and in vivo [21, 26–28]. PEDF regulates vascular permeability by preventing the dissociation of AJ and TJ proteins and regulating AJ protein phosphorylation via γ-secretase pathway [29]. PEDF has also been reported to prevent increased vascular permeability caused by hypoxia stress [21] and protect ox-LDL-induced endothelial cell damage by inhibiting the Wnt signaling pathway [30]. However, it is unknown whether PEDF could repair the damaged endothelium and promote hematopoietic reconstruction during preconditioning process. Our study aims to investigate the effect of PEDF on injured endothelial repair and hematopoietic reconstruction during preconditioning to provide new ideas for reducing PGF and accelerating hematopoietic reconstruction.

## Methods

### Cell culture

bEnd-3 (ATCC® CRL-2299™) were used between the fourth and tenth passage and cultured in Dulbecco’s Modified Eagle Medium (DMEM, Gibco, catalog number: C11995500BT) supplemented with 10% fetal bovine serum (FBS, Gibco, catalog number: 10099–141).

### Irradiation injury cell model and grouping

Endothelial cells (EC) (1 × 10^5^ per well in 6-well plate) received irradiation using GSR C1 137 caesium gamma irradiator (Gamma-Service Medical, Bautzner, Germany) at a dose of 15Gy with a dose rate of 1.88 Gy/min and cultured at 37 °C incubator for 72 h.

PEDF + 15 Gy EC group: 6 h before irradiation, endothelial medium containing recombinant mouse PEDF protein(100 ng / ml, sangon biotech, China) was added into cells followed by receiving irradiation.

PEDF + Atglistatin group: ATGL inhibitor Atglistatin (10 μM, MedChemExpress, CAS No.: 1469924–27-3) was added 1 h before PEDF administration and 6 h before irradiation, endothelial medium containing PEDF (100 ng/ml) was added followed by receiving irradiation.

### Primary HSC culture

Mouse bone marrow cells (BMC) were collected using Lin-sorting (EasySep™ Mouse Hematopoietic Progenitor Cell Isolation Kit, STEM CELL, Catalog # 19856) and CD117 sorting (CD117 MicroBeads, MACS, Cat # 130–091-224). Endothelial cells were inoculated in 24-well plates at a density of 2 × 10^4^/ml per well. After adherence for 24 h, endothelial cells were damaged by Busulfan (0.06 μg/μl). The next day, isolated bone marrow cells (BMC) was inoculated according to the optimal culture ratio (b3: HSC = 1: 6) and cultured. Primary HSC medium consisted of Stem Span™ SFEM (STEM CELL, catalogue number: 09650), SCF (20 ng / ml) (Peprotech, catalog number: 250–03), TPO (20 ng / ml) (Peprotech, catalogue number 315–14), Pen Strep (Gibco, catalogue number: 15140–122). PEDF (100 ng / ml) was added into cocultured medium.

### Experimental animals

SPF male inbred C57BL/6 mice aged 6–8 weeks were purchased from Beijing Weitong Lihua Experimental Technology Co., Ltd. and raised at Experimental Animal Center. This study was approved by the Animal Ethics Committee of Xuzhou Medical University.

### qRT-PCR

RNA was isolated from endothelial cells followed by cDNA synthesis for measuring the expressions of occludin and Zona occludens proteins 1 (ZO-1) by qRT-PCR. The primer sequences were as follows:

Occludin-F-5′-TTGAAAGTCCACCTCCTTACAGA-3′; Occludin-R-5′-CCGGATAAAAAGAGTACGCTGG-3′. ZO-1-F-5′-GCTTTAGCGAACAGAAGGAGC-3′; ZO-1-R-5′-TTCATTTTTCCGAGACTTCACCA-3′.

PEDF-F-5′-GCCCTGGTGCTACTCCTCT-3′;

PEDF-R-5′-CGGATCTCAGGCGGTACAG-3′.

β-actin-F-5′-ATGTGGATCAGCAAGCAGGA-3′; β-actin-R-5′-AAGGGTGTAAAACGCAGCTCA-3′.

### PEDF-siRNA transfection

PEDF shRNA lentiviral plasmids were constructed using GV248 plasmid (hU6-MCS-Ubiquitin-EGFP-IRES-puromycin, Genechem) the sequences of Easy-siRNA are designed as follows, the oligo synthesis information of PEDF-shRNA (88706–1, sh6): 5′-CCGGGCCCAGATGAAAGGGAAGATTCTCGAGAATCTTCCCTTTCATCTGGGCTTTTTG-3′; 5′-AATTCAAAAAGCCCAGATGAAAGGGAAGATTCTCGAGAATCTTCCCTTTCATCTGGGC-3′.

Sh6 targeted sequence was GCCCAGATGAAAGGGAAGATT.

PEDF-shRNA (88707–1, sh7): 5′-CCGGGGCCATCTTACGATACGGCTTCTCGAGAAGCCGTATCGTAAGATGGCCTTTTTG-3′; 5′-AATTCAAAAAGGCCATCTTACGATACGGCTTCTCGAGAAGCCGTATCGTAAGATGGCC-3′.

Sh7 targeted sequence was GGCCATCTTACGATACGGCTT.

Control (shctrl) sequence was TTCTCCGAACGTGTCACGT. Lentivirus were prepared followed the protocol of Genechem. Endothelial cells were cultured in antibiotic-free growth medium to reach about 50% confluence and infected following the company’s experimental procedures of Genechem. Positive cells were selected using puromycine.

### Western blot

The expressions of occludin were detected in endothelial cells by western blot. Total protein was isolated, separated on 10% SDS-PAGE, and transferred to PVDF membrane. After blocking with 5% BSA for 2 h, the membrane was incubated with the primary antibodies against occludin (1: 1000, servicebio, catalogue number: GB11149–2), GAPDH (1: 1000, servicebio, catalogue number: GB11002) at 4 °C overnight followed by washing with TBST and incubation with HRP-labeled goat anti-rabbit secondary antibody (1: 5000, servicebio, catalogue number: GB23303) for 1 h. The membrane was developed after addition of ECL reagent.

### HE staining

Mice were sacrificed and femurs were isolated. After fixation decalcification, dehydration and paraffin embedding, femurs were sliced into 4 μm thickness and stained with HE Solutions. The pathologic changes of bone marrow were estimated by the optical microscope (Nikon).

### Immunofluorescence staining

Cells or frozen sections were fixed in 4% paraformaldehyde for 1 h, blocked with 5% BSA containing 0.3% Triton X-100 for 1 h, and incubated with primary antibody against CD144 (1: 500, Abcam, catalogue number: ab205336) at 4 °C overnight followed by washing 3 times with PBS and incubation with anti-rabbit IgG (H + L), F (ab ‘) 2 Fragment (Alexa Fluor® 488 Conjugate) (1: 500, CST, catalogue number: 4412S) for 2 h. After washing in PBS three times, DIPA (1 mg / ml) was added for 10 min incubation followed by washing twice with PBS, mounting on an anti-tissue fluorescence quencher and observation under a microscope.

Mice femurs were isolated and keeped in 4% PFA for 2 h at 4 °C. After decalcification and dehydration, the femurs were embedded with 8% gelatin 20% sucrose 2% PVP and kept at − 80 °C. Femurs were sliced into 20–30 μm thickness. Non-specific binding were blocked with 4%BSA in PBS at room temperature for 1 h. The desired primary antibody diluted in PBS with 1%BSA were covered the sections at 4 °C overnight. For bone marrow endothelial staining, we used Mouse Endomucin Antibody (RD, AF4666). Rat anti-CD150 (Biolegends, 115,902), Rat anti-CD48 (Biolegends, 103,432), Rat anti-lineage-biotin (Biolegends), DyLight® 550 (ThermoFisher, SA5–10027) and Streptavidin-Brilliant Violet 421™ (Biolegends, 405,225) were used for HSC staining.

### Cell colony formation

Prepare a cell suspension that is 10× the final plating concentration (2 × 10^5^ cells/mL for 2 × 10^4^ cells / dish) by diluting with Iscove’s MDM (STEM CELL, catalogue number:07700) + 2% FBS. Add 300 μL of cells to 3.0 mL of MethoCult™ (STEM CELL, catalogue number: 03444) for duplicate dishes. The contents were mixed thoroughly. Let stand for at least 5 min to allow bubbles to rise to the top. Dispense MethoCult™ mixture into 35 mm dishes for methylcellulose-based cultures using a blunt-end needle attached to a syringe. Place the culture dishes in a large outer dish containing uncovered 100 mm dishes filled with sterile water to maintain proper humidity of MethoCult™ medium. Incubate cells for 10 days in a humidified incubator at 37 °C and 5% CO_2_.

### Cell cycle determination

2 μl each of the corresponding Lin strains markers (Ter119, CD45R / B220, CD11b, CD8a, CD4, Gr-1), C-kit and Sca-1 was added to the cell suspension and incubated at 4 °C for 25 min followed by washing and addition of FIX & PERM® Sample Kit A (Nordic-MUbio, catalogue number: GAS-002 M) for 15 min incubation and subsequent addition of 100 μl of FIX & PERM® Sample Kit B (Nordic-MUbio, catalogue number: GAS-002 M) and 30 μl DIPA for overnight incubation at 4 °C. Then, cell cycle was analyzed.

### Flow Cytometry

Bone marrow cells were flushed from the femurs and tibias of mice with PBS containing 2% FBS and lysed with lysing buffer (catalogue number: R1010, Solarbio, China) for red blood cell deletion. Bone marrow cells were suspended in PBS and stained by antibodies for 30 min. For Detection lineage-specific markers: Erythroid: Ter119 (FITC, Biolegend, catalogue number: 116206) and CD71 (PE, eBioscience, catalogue number:12–0711-82), Granular line: CD11b (FITC, BD, catalogue number:557396) and Gr-1 (PE, BD, catalogue number: 553126), Megakaryocyte: CD41 (FITC, BD, catalogue number:553848) and C-kit (PE, BD, catalogue number:553355), LSK: lineage cocktail (FITC), sca-1 (APC, Biolegend, catalogue number:108112) and C-kit (PE, BD, catalogue number:553355). Cell apoptosis analysis was performed using Annexin V staining kit according to the instructions of the manufacturer (BD).

### Cell permeability assessment

Cells were seeded in the Transwell chamber (200 μl) at a density of 2 × 10^4^/ml. After 72 h, the upper and lower cells were washed with PBS, 200 μl of FITc-Dextran (1 mg / ml) was added to the upper chamber and 200 μl PBS was added to the lower chamber for 3 h incubation. 100 μl of liquid was collected from the lower chamber for measuring the optical density (OD) value. Pa = [A] / t × (1 / A) × (v / [L]), where [A] is the FITC-Dextran concentration in the top chamber (expressed as fluorescence intensity), t is time (sec), A is the area of the filter membrane (cm^2^), and v is the amount of liquid in the bottom chamber (ml), [L] is FITC-Dextran concentration in the bottom chamber (expressed as fluorescence intensity). The results were expressed as the percentage change of Pa, Pa% = (Experimental group Pa) / (Control group Pa) × 100%.

### BMT model

BMT mice were divided into PEDF + BMT group and BMT group and samples were collected at day 7, 14, 21, and 28 after bone marrow cell transplantation. Both groups of mice received an irradiation of 8.5 Gy. PEDF group received intraperitoneal injection of PEDF recombinant protein at 0.5 mg/kg 12 h before irradiation and every 2 days afterwards. The BMC group received intraperitoneal injection of the same amount of PBS. The next day after receiving radiation, bone marrow cells of syngeneic mice were taken for HSC transplantation through injection of 5 × 10^6^ bone marrow cells via tail vein.

### ELISA

ELISA kits were used to detect the levels of SCF, TGF-β, IL-6 and TNF-α in the cell culture supernatant and bone marrow supernatant.

### Statistical analysis

All data were analyzed by GraphPad Prism 8.0 software and expressed as mean ± standard deviation (SD). Comparisons between two groups were performed by t-test and analysis of variance was used for comparison between multiple groups. *P* < 0.05 indicates a significant difference.

## Results

### Injured endothelial cells affect HSC expansion

**Fig. 1:**
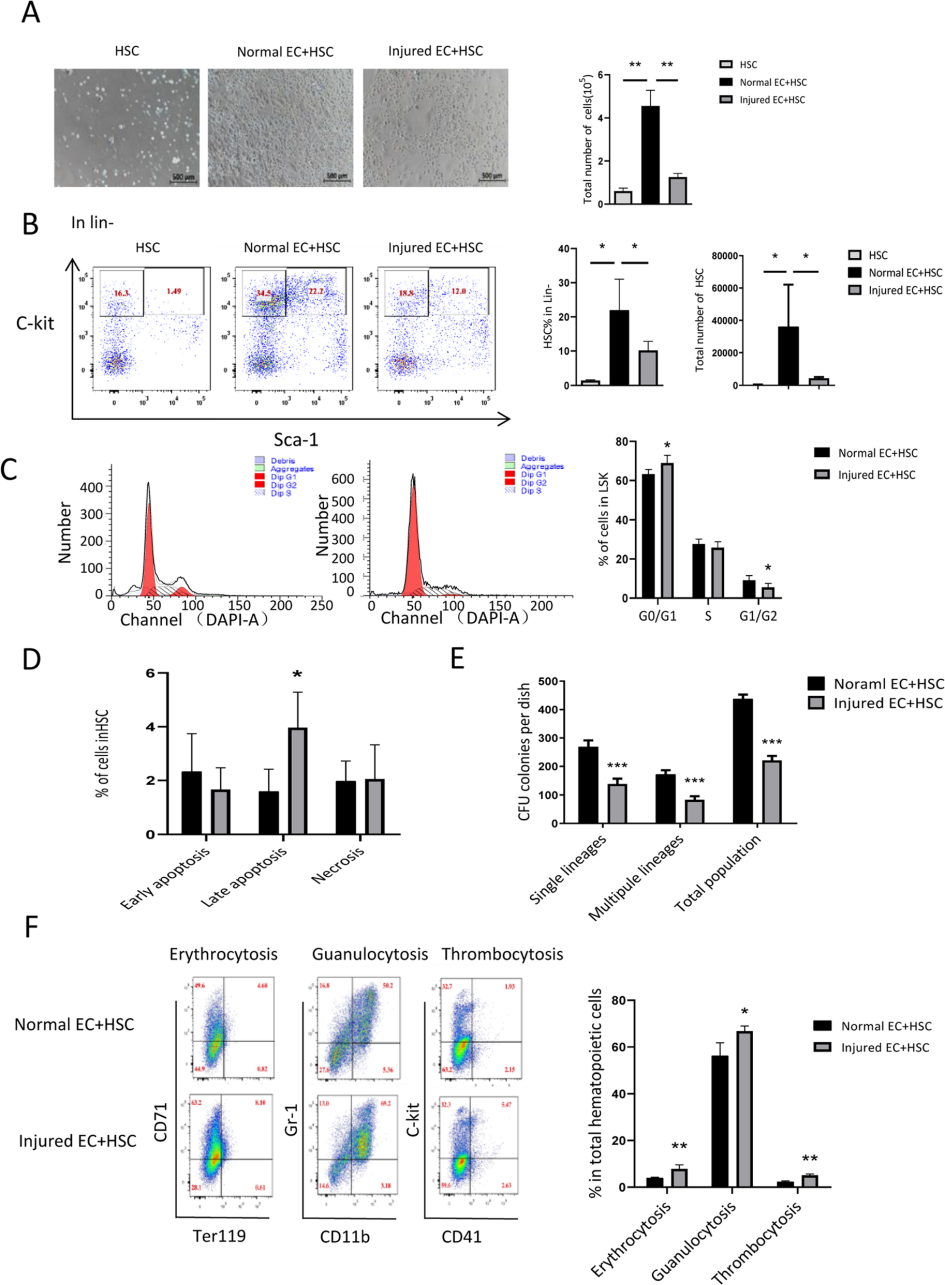
In vitro analysis of the effect of injured EC on HSC expansion, cell cycle, apoptosis and differentiation in a co-culture system. **a** Representative Microscopic observation of HSC expansion after cocultured with normal or damaged endothelial cells for 7 days; **b** Flow cytometry analysis of the proportion and number of HSCs after co-culture with normal endothelial cells or damaged endothelial cells. **c** HSC cell cycle analysis by flow cytometry; **d** HSC apoptosis analysis; **e** Colony-forming capacity of HSCs after co-culture; **f** Flow cytometry analysis of the proportion of erythrocytes, granulocytosis and thrombocytosis (*n* = 3–4, * *P* < 0.05, ** *p* < 0.01, *** *p* < 0.0001)

A large number of studies have demonstrated that bone marrow microvascular endothelium is severely damaged after HSCT, affecting hematopoietic reconstruction. However, the effect of damaged endothelial microenvironment on HSC is unknown. To investigate this, our study first established an in vitro co-culture model of Busulfan injured endothelial cells and HSC. It is known that vascular endothelial cells, as important hematopoietic stromal cells, can promote HSC proliferation which was confirmed by our study (Fig. 1a). Meanwhile, we found that co-culture of damaged endothelial cells and HSC inhibited HSC expansion (Fig. 1a), leading to a decrease in the proportion and total number of HSC (Fig. 1b). In addition, the ratio of G0 / G1 phase of HSC in co-culture group was increased, leading to reduced HSC proliferation (Fig. 1c) and increased apoptosis (Fig. 1d). Further analysis showed that the colony-forming ability in co-culture group was reduced (Fig. 1e). Consistently, the percentage of red blood cells, granulocytes, and megakaryocytes in the co-cultured group was increased significantly (Fig. 1f). The above results suggest that damaged endothelial cells inhibit HSC expansion in vitro.

### PEDF protects preconditioning-induced injury of endothelial cells in vitro

**Fig. 2:**
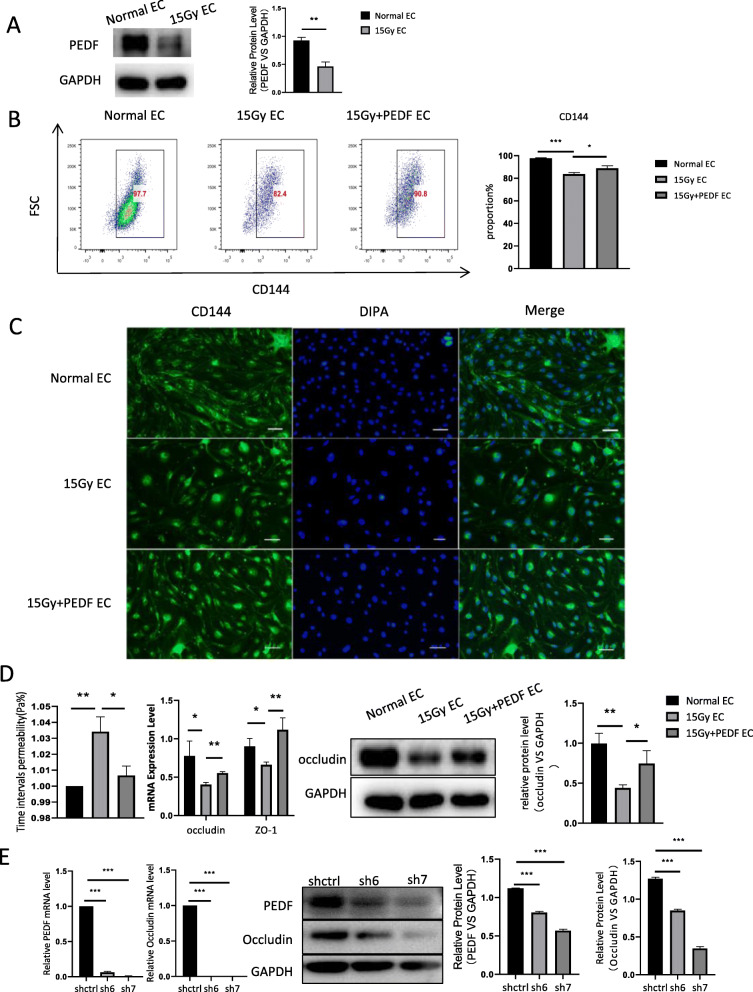
PEDF improves endothelial permeability. **a** Detection of PEDF level in irradiation injured endothelial cells by western blot. **b** Flow cytometry detected endothelial cell surface marker CD144 expression after irradiation. **c** Cell immunofluorescence (× 100) analysis of CD144 expression. **d** Transwell assay analysis of endothelial cell permeability and the expression of occludin and ZO-1. **e** PEDF shRNA (sh6 and sh7) were used to knock down PEDF and check the occludin expression in injured endothelial cells after irradiation (*n* = 3, * *p* < 0.05, ** *p* < 0.01, *** *p* < 0.001)

We found that the level of pigment epithelium-derived factor (PEDF) was decreased significantly after endothelial cell injury byγ-irradiation (Fig. 2a) without changes of vascular endothelial growth factor (VEGF) and platelet derived growth factor PDGF (data not shown). As PEDF is an endogenous functional protein with cytoprotective effects and closely related to endothelial cell permeability. Therefore, we tested whether PEDF has a protective effect on the damaged endothelial cells and found that CD144 expression was significantly reduced after irradiation and increased after addition of PEDF (Fig. 2b). Consistently, immunofluorescence assay also showed the increased expression of CD144 in endothelial cells after treatment with PEDF (Fig. 2c). In addition, further analysis confirmed that PEDF can inhibit the increase of endothelial permeability which is caused by irradiation and elevate the expression of occludin and ZO-1, which are endothelial permeability related indicators (Fig. 2d). We also used PEDF shRNA to knock down PEDF expression to check PEDF’s effect on permeability in the irradiation cell model and found that PEDF knock-down could inhibit the occludin mRNA and protein expression (Fig. 2e).

### PEDF improves the permeability of endothelial cells after irradiation through ATGL receptors

**Fig. 3:**
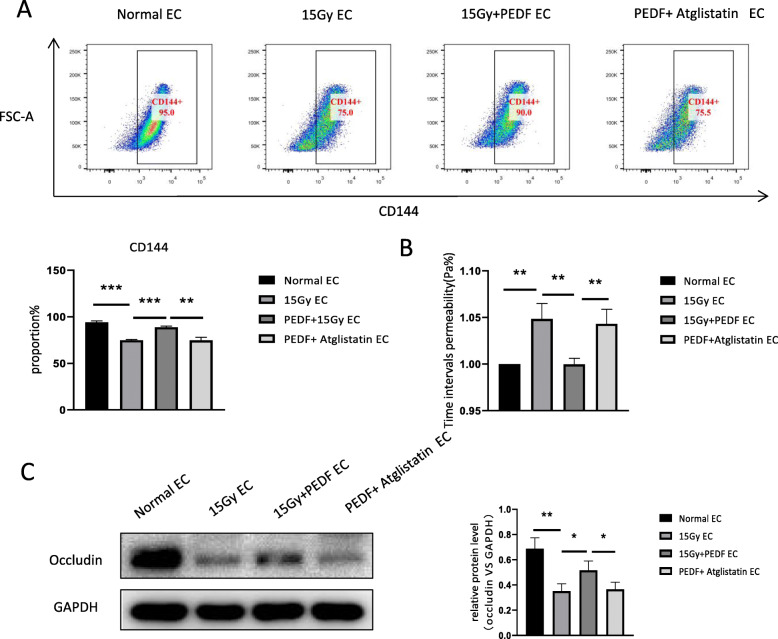
PEDF protects endothelial permeability through the ATGL receptor. **a** Flow cytometric analysis of CD144 expression levels after addition of the PEDF receptor inhibitor Atglistatin. **b** Endothelial cell permeability analysis in the presence of Atglistatin; **c** WB analysis of the expression of endothelial cell junction molecule occludin after addition of PEDF receptor inhibitor Atglistatin (*n* = 3, * *p* < 0.05, ** *p* < 0.01, *** *p* < 0.001)

To determine whether PEDF receptor Adipose triglyceride lipase (ATGL) is involved in PEDF’s effect on the permeability of injured endothelial cells, we used the ATGL inhibitor Atglistatin and found that the increase of CD144 expression induced by PEDF was suppressed after addition of Atglistatin (Fig. 3a). In addition, Atglistatin can abolish the protective effect of PEDF on the permeability of injured endothelial cells (Fig. 3b). Moreover, the increase of occludin expression induced by PEDF was also inhibited (Fig. 3c). This data show that ATGL receptors are involved in the regulation of PEDF’s effect on injured endothelial cell permeability.

### PEDF ameliorates the inhibitory effect of damaged endothelial cells on HSC expansion

**Fig. 4:**
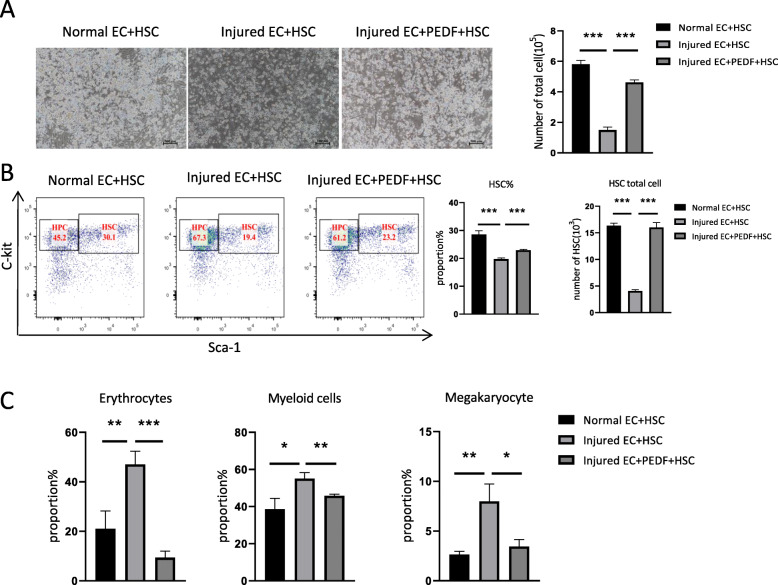
PEDF protects endothelial injury from the function-inhibiting effect of hematopoietic stem cells. **a** Representative microscopic observation of HSC expansion after cocultured with normal or damaged endothelial cells for 7 days with or without PEDF (× 100) and total number of cultured HSC analysis. **b** Flow cytometry detection of HSC proliferation. **c** Flow cytometry analysis of the proportion of erythrocytes, myeloid cells and megakaryocytes (*n* = 3, * *P* < 0.05, ** *p* < 0.01, *** *p* < 0.001)

In order to determine whether PEDF can reverse the inhibitory effect of injured endothelial cells on HSC, PEDF was added to the co-culture system and our results showed that PEDF can significantly improve the reduced HSC expansion ability induced by Busulfan damaged endothelial cells (Fig. 4a). Meanwhile, PEDF can increase the reduction of HSC% and restore the number of HSCs in the co-culture system (Fig. 4b). Additionally, addition of PEDF can significantly inhibit the differentiation of HSC induced by damaged endothelial cells (Fig. 4c). The above results suggest that PEDF can ameliorate the inhibitory effect of injured endothelial cells on HSC and promote HSC expansion in vitro.

### PEDF accelerates hematopoietic reconstitution after BMT

**Fig. 5:**
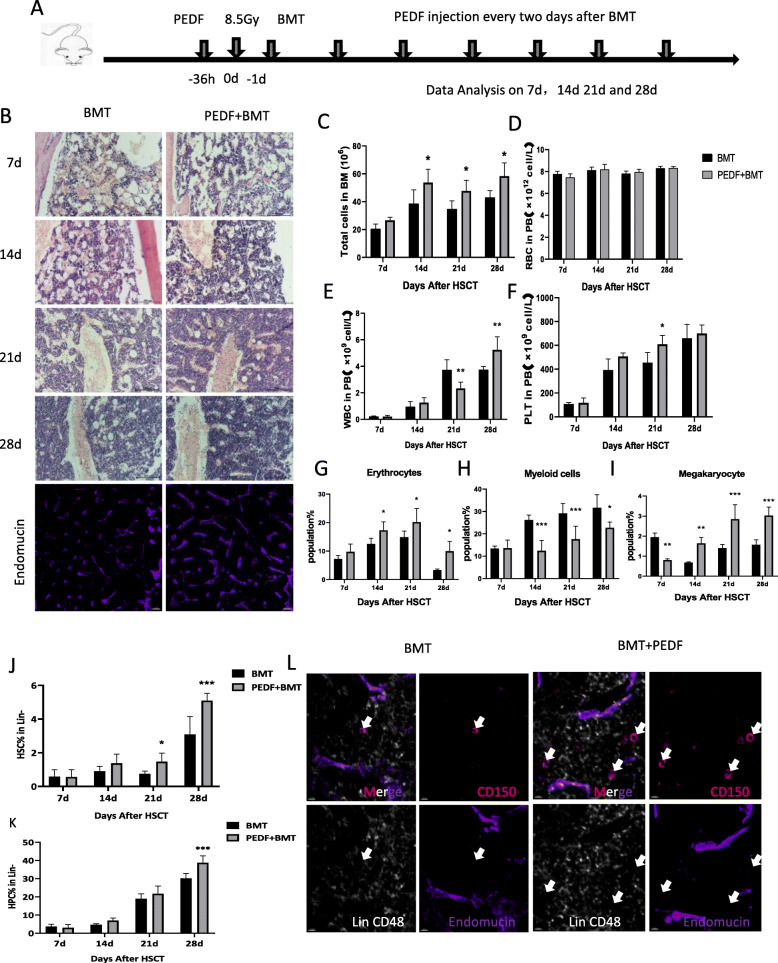
PEDF improves hematopoietic reconstruction after BMT. **a** Schematic graph of BMT experiments; **b** Bone marrow pathology analysis using HE staining on d7, d14, d21 and d28 and immunostaining on d28; and **c** Bone marrow cell (BMC) count; **d**-**f** Peripheral blood cell count including white blood cells (WBC), Red blood cells (RBC) and Platelets (PLT). **g**-**i** Flow cytometry analysis of the proportion of erythrocytes, myeloid cells and megakaryocytes. **j**, **k** The proportion of HSC and HPC analyzed by flow cytometry; **l** Representative images of a bone marrow section showing Lin-CD48-CD150+ hematopoietic stem cell and Endomucin+ bone marrow endothelial cell staining (*n* = 3, * *P* < 0.05, ** *p* < 0.01, *** *p* < 0.001)

To determine whether PEDF can promote hematopoietic reconstruction after BMT, mouse PEDF recombinant protein was injected before and after BMT (Fig. 5a) and specimens were collected on day 7, 14, 21 and 28 after transplantation. Bone marrow pathology analysis on day 7, 14,21,28 suggested that hematopoietic recovery was gradually recovered after transplantation, while PEDF could reduce bone marrow pathological and endothelial damage (Fig. 5b) and accelerate hematopoietic reconstruction as demonstrated by improve recovery of the number of white blood cells and platelets (Fig. 5d-f). Bone marrow cell count results also showed that the number of bone marrow cells in the PEDF + BMT group was significantly higher than that in the BMT group at 14 days, 21 days, and 28 days after transplantation (Fig. 5c). Moreover, PEDF increased the proportion of erythrocytes and megakaryocytes and decreased myeloid cells (Fig. 5g-i). Furthermore, the proportion of hematopoietic stem progenitor cells (HSPC) was also significantly higher after PEDF addition (Fig. 5j and k). The immunostaining analysis of Lin-CD48-CD150+ HSC and bone marrow further suggest that PEDF promotes hematopoietic stem cell reconstruction (Fig. 5l) and the repair of bone marrow endothelial injury (Fig. 5b) after BMT.

### PEDF promotes the secretion of SCF, TGF-β and IL-6

**Fig. 6:**
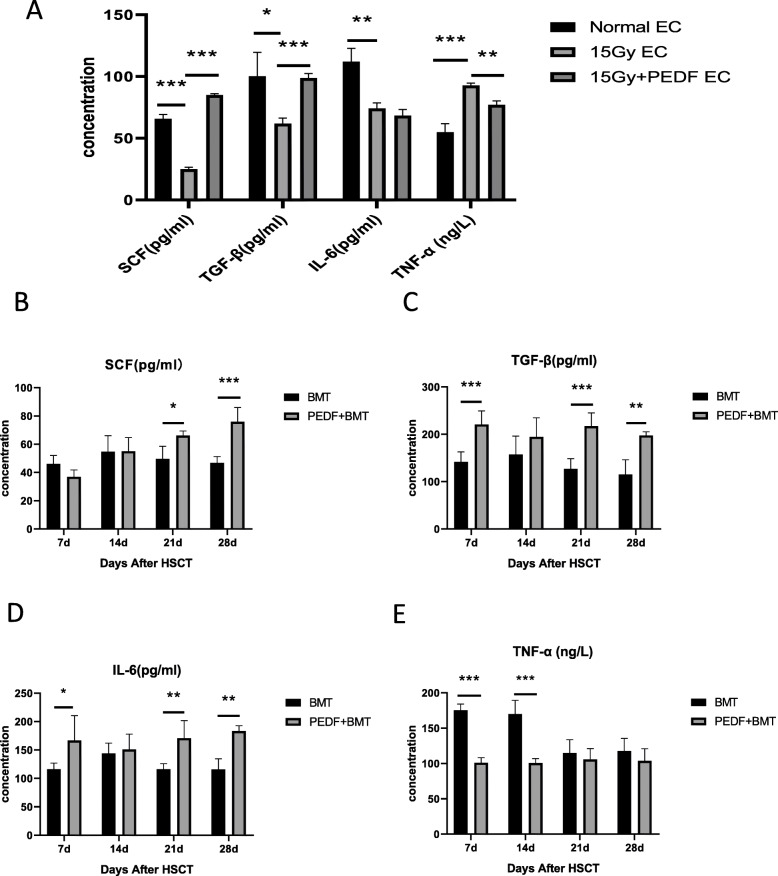
Effect of PEDF on the secretion of hematopoietic related factors. **a** In vitro experiments analysis of the levels of SCF, TGF-β, IL-6 and TNF-α by ELISA. In vivo experiments analysis of **b** SCF, **c** TGF-β, **d** IL-6 and **e** TNF-α levels in bone supernatant (*n* ≥ 3, * *p* < 0.05, ** *p* < 0.01, *** *p* < 0.001)

We next tested the levels of hematopoietic related factors SCF, TGF-β, IL-6 and TNF-α and found that the levels of SCF, TGF-β and IL-6 were decreased and TNF-α level was increased after endothelial cells were damaged and their levels were reversed after addition of PEDF (Fig. 6a) in the co-culture system. In vivo experiments further confirmed that PEDF can promote the secretion of SCF, TGF-β, and IL-6 in the bone marrow supernatant of BMT mice and inhibit TNF-α secretion early after transplantation (Fig. 6b-e). The above results suggest that PEDF promotes hematopoietic reconstitution possibly through regulating these cytokines.

## Discussion

Bone marrow microvascular endothelial cells are an important part of the bone marrow microenvironment and play an important role in maintaining bone marrow homeostasis and promoting hematopoietic reconstruction [12, 31, 32]. A large number of data have proved that radiotherapy and chemotherapy can cause severe damage to bone marrow microvascular endothelium [14, 33]. However, there are few reports on the underlying mechanism of endothelial injury and promoting bone marrow endothelial injury repair strategies after radiotherapy and chemotherapy. Our study profoundly elucidates the mechanism of PEDF in repairing damaged endothelial cells and promoting hematopoietic reconstruction.

It is known that endothelial cells are important hematopoiesis-promoting stromal cells and promote the in vitro expansion of hematopoietic stem cells [10]. However, it is unknown whether injured endothelial cells can directly inhibit hematopoietic stem cells. Therefore, this study first established an in vitro microvascular endothelial injury model to study the effect of injured endothelial cells on the biological function of hematopoietic stem cells. We found that damaged endothelial cells can inhibit the expansion of hematopoietic stem cells in vitro, induce cell cycle arrest, apoptosis, and promote the differentiation of hematopoietic stem cells, suggesting that it has a direct inhibitory effect on hematopoietic stem cells. However, how to repair the damaged endothelial cells and ameliorate the effect of damaged endothelium on hematopoietic stem cells is unknown. Therefore, there is an urgent need to explore or discover drugs that can protect the endothelial cells and promote hematopoietic reconstruction.

Pigment epithelium-derived factor (PEDF) is a multifunctional protein secreted by a variety of cells. It has anti-inflammatory, anti-oxidant, anti-fibrotic, anti-vascular permeability enhancement, and cytoprotective effects without toxicities on hematopoietic stem cells [34]. It is a natural treatment strategy for endothelial injury with great potential and promising clinical application. In order to investigate whether PEDF can reverse the inhibitory effect of injured endothelial cells on the biological function of hematopoietic stem cells, we added PEDF to the co-culture system and found that PEDF can reverse the inhibitory effect of injured endothelial cells on hematopoietic stem cells and promote the expansion of hematopoietic stem cells in vitro, suggesting that PEDF has a protective effect on injured endothelial cells and can reverse the inhibitory effect of injured endothelial cells on hematopoietic stem cells.

Next, we found that PEDF injection can promote hematopoietic reconstruction after BMT in mice. Studies have shown that PEDF as a regulator of stem cells can promote the self-renewal of neural hepatocytes [35, 36], but its direct effect on hematopoietic stem cells is minimal, and we have found that PEDF can reduce bone marrow pathological damage and bone marrow microvascular damage. It is suggested that PEDF may repair bone marrow microvessels and then promote hematopoietic reconstruction.

This study also found that the permeability of the injured endothelial cells changed significantly and the PEDF recombinant protein is a secreted protein that is closely related to endothelial permeability. We then assessed the effect of PEDF on injured endothelial function in vitro and found that PEDF can inhibit the permeability changes of injured endothelial cells, so as to maintain endothelial function. Endothelial cell permeability is mainly composed of the paracellular pathway (cell junction) and the transcellular pathway, of which the paracellular pathway is the main pathway [37]. The paracellular pathway mainly has two forms of tight junctions (TJs) and adhesive junctions (AJs). TJ is mainly composed of occludin and claudin. These two types of transmembrane proteins are connected to the actin cytoskeleton through a locking protein (ZO-1) [38]. The increase in vascular permeability caused by TJ is mainly caused by degradation or phosphorylation of related proteins, in which occludin is hydrolyzed to inactive fragments and silk / threonine phosphorylation of ZO-1 protein is closely related to increased permeability [38]. AJs are considered to be the main connection of the peripheral microvascular system. It is mainly composed of the transmembrane protein vascular endothelial cadherin (VE-cadherin), which is fixed on the actin cytoskeleton by catenin (α-, β-, γ-catenin or bridging protein p120) [38]. The disassociation of AJs can lead to increased cell permeability. This study found that the levels of ZO-1, VE-cadherin and occludin in the injured endothelial cells were decreased after irradiation, suggesting that irradiation can increase the permeability of vascular endothelial cells, consistent with previous studies [39, 40]. Mouse bone marrow microvascular endothelium serves as a portal for hematopoietic cells in the bone marrow. The increased microvascular endothelial permeability caused by irradiation may be related to failure implantation of hematopoietic stem cells and bone marrow hemorrhage after irradiation injury in mice. However, PEDF can inhibit the increase of endothelial permeability [21] and promote the colonization of implanted hematopoietic stem cells.

Studies have suggested that PEDF has multiple receptors (PEDF receptor, PEDFR) and it binds to different receptors to play different functions [41–46]. For example, PEDF interacts with the phospholipase A2 receptor, which can exert anti-oxidation and anti-apoptosis or cytoprotection effects; PEDF interacts with laminin receptor (LR) to exert anti-angiogenesis effect and inhibit endothelial cell migration. In addition, the interaction between PEDF and ATGL is related to insulin resistance. However, this study found that the interaction of PEDF with the receptor ATGL might promote the expression of VE-cadherin and occludin, which are related to the permeability of endothelial cells. In addition, this study also found that hematopoietic related factors were changed significantly after addition of PEDF, suggesting that hematopoietic related factors might participate in the effect of PEDF on repairing damaged endothelial cells and promoting hematopoietic reconstruction.

## Conclusions

In conclusion, our study demonstrates that PEDF inhibits the increased endothelial permeability induced by irradiation and reverse the inhibitory effect of injured endothelial cells on hematopoietic stem cells and promote hematopoietic reconstruction.

## Data Availability

All data generated or analysed during this study are included in this published article.

## Abbreviations

BMC: Bone marrow cells
BMT: Bone marrow transplantation
DMEM: Dulbecco’s Modified Eagle Medium
EPC: Endothelial progenitor cell
FBS: Fetal bovine serum
GVHD: Graft-versus-host disease
HSCT: Hematopoietic stem cell transplantation
PGF: Poor graft function
PEDF: Pigment epithelium-derived factor
SCF: Stem cell factor
VEGF: Vascular endothelial growth factor
